# A Multi-Shot Approach for Spatial Resolution Improvement of Multispectral Images from an MSFA Sensor

**DOI:** 10.3390/jimaging10060140

**Published:** 2024-06-08

**Authors:** Jean Yves Aristide Yao, Kacoutchy Jean Ayikpa, Pierre Gouton, Tiemoman Kone

**Affiliations:** 1 Laboratoire Imagerie et Vision Artificielle (ImVia), Université de Bourgogne, 21000 Dijon, France; jean-yves-aristide.yao@u-bourgogne.fr (J.Y.A.Y.); kacoutchy.ayikpa@uvci.edu.ci (K.J.A.); 2 Unité de Recherche et d’Expertise Numérique (UREN), Université Virtuelle de Côte d’Ivoire, 28 BP 536, Abidjan 28, Côte d’Ivoire; president@uvci.edu.ci

**Keywords:** MSFA one-shot, multispectral demosaicing, multi-shot system, spectral resolution, spatial resolution

## Abstract

Multispectral imaging technology has advanced significantly in recent years, allowing single-sensor cameras with multispectral filter arrays to be used in new scene acquisition applications. Our camera, developed as part of the European CAVIAR project, uses an eight-band MSFA to produce mosaic images that can be decomposed into eight sparse images. These sparse images contain only pixels with similar spectral properties and null pixels. A demosaicing process is then applied to obtain fully defined images. However, this process faces several challenges in rendering fine details, abrupt transitions, and textured regions due to the large number of null pixels in the sparse images. Therefore, we propose a sparse image composition method to overcome these challenges by reducing the number of null pixels in the sparse images. To achieve this, we increase the number of snapshots by simultaneously introducing a spatial displacement of the sensor by one to three pixels on the horizontal and/or vertical axes. The set of snapshots acquired provides a multitude of mosaics representing the same scene with a redistribution of pixels. The sparse images from the different mosaics are added together to get new composite sparse images in which the number of null pixels is reduced. A bilinear demosaicing approach is applied to the composite sparse images to obtain fully defined images. Experimental results on images projected onto the response of our MSFA filter show that our composition method significantly improves image spatial resolution and minimizes reconstruction errors while preserving spectral fidelity.

## 1. Introduction

Multispectral images are useful in a wide range of applications: facial recognition [1], remote sensing [2], medical imaging [3], and precision agriculture [4], among others. Multispectral image acquisition systems offer great diversity, particularly with scanning mode acquisition systems that acquire the multispectral image in multiple frames. They are divided into three categories: tunable filter cameras, tunable illumination cameras, and multi-camera systems. Tunable filters, such as LCTF (Liquid Crystal Tunable Filter) [5] and AOTF (Acousto-Optical Tunable Filter) [6], use electronic techniques to capture each multispectral band. Although these systems produce fully defined multispectral images, their acquisition time is beyond the scope of a real-time acquisition system.

On the other hand, instantaneous acquisition systems, or snapshots, capture the MS image in a single shot. They include single-sensor or multi-sensor multispectral systems, which are divided into several classes: multispectral filter array (MSFA), interferometers, tunable sensors, and filtered lens arrays [7].

The acquisition system based on a single-sensor one-shot camera coupled with an MSFA provides a compact, low-cost, real-time solution for multispectral image acquisition. The camera can capture all the necessary spectral bands in a single snapshot [8]. To achieve this, an MSFA is positioned in front of the sensor to capture mosaic images where each pixel location contains information from a single spectral band. An interpolation method is applied to the mosaic image to obtain the fully defined multispectral image [9].

The MSFA plays a crucial role in multispectral imaging by filtering the light entering the sensor. In a defined MSFA, the number of bands increases by reducing the number of pixels assigned to the band. A greater number of spectral bands in the MSFA allows for a more precise spectral analysis of the observed scene, but this results in a decrease in the spatial resolution of the image. Indeed, with more spectral bands, the distance between spectrally similar pixels increases [10]. The main weakness of single-sensor one-shot cameras is their ability to efficiently reconstruct a complete multispectral image from a mosaic image, especially when the mosaic contains non-homogenous areas, abrupt transitions, and textured regions [11].

Previous works [4,12] have detailed our single-shot multispectral camera’s design process, specifically designed to operate in the visible. This camera has a 4 × 4 MSFA moxel with eight spectral bands selected by a genetic algorithm. Each spectral band receives two pixels per moxel, where the mosaic arrangement is a moxel assembly over a monochrome sensor [13]. After a snapshot, the camera provides a mosaic that is decomposed into eight sparse images, each containing pixels with the same spectral properties and null pixels. Thus, the sparse images have a very high number of null pixels. For our camera, in a 16-pixel moxel window, 14 pixels are null. This deficit can cause problems during image demosaicing, affecting image quality and visual fidelity and a loss of spatial resolution.

To address these issues, we propose a method for reducing the number of null pixels in sparse images. Our approach aims to reduce the number of null pixels by combining sparse images from multiple acquisitions. To achieve this, we combine camera displacements along both the vertical and horizontal axes. At each displacement, the camera captures an image of the observed scene, generating a mosaic of the scene with a spatial redistribution of pixels with similar spectral properties. Next, the set of sparse images from each post-displacement acquisition is summed with those obtained without displacement to obtain new composite sparse images. The new sparse images are finally demosaiced.

In this study, we present the following contributions:

- Setting up a dataset for our experiments, consisting in transforming images from a database of 31 into 8 bands to simulate our 8-band MSFA moxel. These images will then be mosaicked with our MSFA filter to simulate a snapshot from our camera;
- Development of a new composition method with a multi-shot approach to reduce the number of null pixels in sparse images while maintaining the same number of spectral bands;
- Performed visual and analytical comparisons using validation metrics to evaluate our experiments, demonstrating the improvement in spatial resolution of the final image obtained after demosaicing.

The remainder of this article is organized as follows: Section 2 presents the state of the art in improving the spatial resolution of MSFA images. Section 3 details the materials and methods used in our approach. Section 4 presents the experiments carried out and the results obtained. Section 5 discusses the results. Finally, Section 6 presents our conclusion.

## 2. Related Works on Improving the Spatial Resolution of MSFA Images

Much research has demonstrated the interest in improving the spatial resolution of multispectral images from an MSFA sensor.

Monno et al. [11,14] proposed a multispectral demosaicing method using a guided filter. This method is used in multispectral imaging to improve color reproduction and computer vision applications. The proposed method uses a guided filter to interpolate spectral components in a multispectral color filter array. The technique addresses the challenge of undersampling in multispectral imaging and shows promising results for practical applications. Its effectiveness is based on the establishment of an MSFA pattern with a dominant green band.

Wang et al. [15] proposed a method to improve the quality of images reconstructed from multispectral filter networks while minimizing the computational cost. It addresses the challenge of estimating missing data in images acquired by these networks using adaptive frequency domain filtering (AFDF). This technique combines the design of a frequency domain filter to eliminate artifacts with spatial averaging filtering to preserve spatial structure. By incorporating adaptive weighting, AFDF improves the quality of reconstructed multispectral images while maintaining high computational efficiency.

Rahti and Goyal [16] proposed a weighted directional interpolation method for estimating missing pixel values. They exploit both spectral and spatial correlations present in the image to intelligently select interpolation schemes based on the properties of binary tree-based MSFA models. By computing directional estimates and using edge amplitude information, the method progressively estimates missing pixel values and updates pixel arrangements according to the band’s point of arrival (PoA) in the binary tree structure.

Zhang et al. [17] proposed a method that integrates a deep convolutional neural network with a channel attention mechanism to improve the demosaicing process. In this method, a mean square error (MSE) loss function is used to improve the accuracy of estimated pixel values in image processing. In addition, a contour loss is introduced to improve the sharpness and richness of textured images using high-frequency subband analysis in the wavelet domain. The method uses the TT-59 database [18] for training and evaluation. Multispectral images are processed to synthesize radiance data to demonstrate the effectiveness of the demosaicing technique.

Mihoubi et al. [19] proposed a demosaicing method called PPID based on the generation of a pseudo-panchromatic image (PPI). To ensure robustness to different lighting conditions, an adjustment of the value scale in the raw image is proposed before estimating the PPI, with the aim of mitigating biases caused by differences in spectral illumination distribution between channels. The remaining steps include calculating the spectral differences [20] between the original raw image and the PPI, using local directional weights for interpolation [21], and, finally, combining the PPI with the differences to estimate each channel of the final image.

Jeong et al. [22] proposed a method to improve image quality by estimating a pseudo-panchromatic image using an iterative linear regression model. It then performs directional demosaicing, a technique that combines the pseudo-panchromatic image with spectral differences to produce a final interpolated image. The process includes steps such as directional interpolation using the BTES method [23] and calculation of weights to improve the accuracy of the final multispectral image.

Rathi and Goyal [9] proposed a method that uses the concept of the pseudo-panchromatic image and spectral correlation between spectral bands to efficiently generate a complete multispectral image. It involves estimating a pseudo-panchromatic image from a mosaic image using convolution filters based on the probability of the appearance of each spectral band [24] and binary masks. This pseudo-panchromatic image is then used to interpolate each spectral band to produce a multispectral image. The process iteratively improves the quality of the multispectral image by updating the pseudo-panchromatic image and estimating the spectral bands multiple times.

Liu et al. [25] proposed a new deep learning framework for multispectral demosaicing using pseudo-panchromatic images. The framework consists of two networks, the Deep PPI Generation Network (DPG-Net) and the Deep Demosaic Network (DDM-Net), which are used to generate and refine the PPI to improve image quality and recover high-frequency information in the demosaicing process. DPG-Net specifically focuses on improving the sharpness of the preliminary PPI to improve image resolution by learning the differences between the actual PPI and Mihoubi’s blurred version [19], which ultimately leads to the production of the final refined PPI. DDM-Net uses bilinear interpolation to estimate missing pixel values in fragmented bands, followed by a neural network architecture that extracts color and texture features to improve image quality. By combining convolutional layers and loss functions, DDM-Net aims to minimize reconstruction errors and produce high-quality demosaiced images.

Zhao et al. [26] proposed a neural network model with two branches of adaptive features (DDMF) and edge infusion (PPIG). The proposed architecture combines weighted bilinear interpolation [21] to generate initial demonstration images with adaptive adjustments of pixel values in reconstructed multispectral images. It uses a DDMF module to generate convolution kernel weights that adapt to spatial and spectral changes, thus improving the accuracy of the demosaicing process. In addition, the PPIG edge infusion sub-branch integrates edge information to improve demosaicing accuracy in terms of spatial precision and spectral fidelity.

Most of the methods proposed to improve the spatial resolution of a multispectral image are based on complex steps during the demosaicing process. Our paper proposes a new approach based on a multi-shot method that happens before the demosaicing process.

## 3. Materials and Methods

### 3.1. The MSFA Moxel

The MSFA moxel is a grid of optical filters placed in front of the sensor of a multispectral camera to filter the incoming light into different spectral bands. Each pixel in the captured image is associated with a specific filter in the MSFA moxel, allowing light intensity to be measured in different parts of the electromagnetic spectrum. The MSFA allows the simultaneous acquisition of multispectral information during image acquisition by distributing the pixels on the image sensor according to their spectral sensitivity. The choice of MSFA size and the number of bands is essential for the acquisition and reconstruction of multispectral images. The MSFAs commonly used in the literature generally have the following two main characteristics:

- Redundancy [27]: a band can have a probability of appearance greater than one, 1n, where n represents the linear size of the MSFA moxel;
- Non-redundancy [21]: each band has a probability of appearance of 1n.

In the case of bands with redundancy, the following two types of behavior can be observed:

- Dominant bands: the probability of the appearance of certain bands in the MSFA moxel is higher than others;
- Nondominant bands: all bands in the MSFA moxel have the same probability of appearance.

These characteristics of the MSFA moxel directly affect the quality and resolution of the multispectral images obtained after the acquisition and reconstruction process. The selection of the appropriate MSFA moxel depends on the specific application requirements, such as the desired spectral resolution, sensitivity to different wavelengths, and camera hardware constraints.

**Figure 1:**
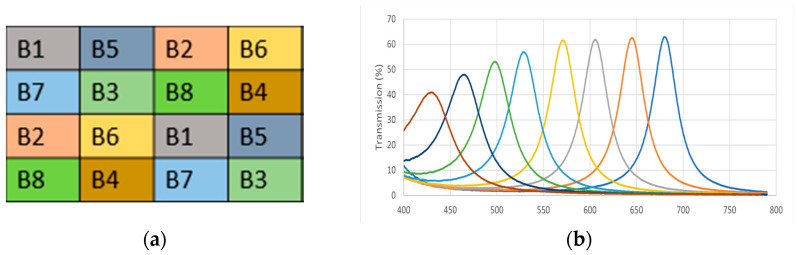
(**a**) A 4 × 4 size nondominant band redundant MSFA moxel. (**b**) Spectral response of the MSFA moxel of the CAVIAR project camera.

Our camera uses a 4 × 4 filter with equal probability of band appearance to acquire mosaic images, where each band is sampled by two pixels. This moxel was chosen to balance the spatial distribution of pixels in sparse images [28]. This design is based on the color shade approach [12], which optimizes the spectral response of the filters and improves the quality of images acquired during a shot. Figure 1a illustrates the spectral band arrangement of our MSFA moxel. This moxel is used throughout our study to construct mosaic images and in demosaicing multispectral images.

Figure 1b shows the filters’ spectral response in our MSFA model. Spectral response refers to how well the sensor detects and measures light in different spectral bands. This spectral response is given in the visible spectral interval [400 nm, 790 nm].

### 3.2. Dataset

In our simulation, we project 31 image bands from the TokyoTech database (TT-31) [11] into 8 bands corresponding to our MSFA. This projection is performed on the response of the MSFA filters of our camera. The use of this projection is important because it allows us to work with accurate data that reflect the conditions we encounter in the real world when making acquisitions with our camera. This allows us to reduce the dimensionality of the images while preserving the most relevant spectral information. Here are the steps in the projection process:

- Determination of the desired number of bands for the resulting multispectral image, in our study, eight bands.
- Definition of Gaussian filter full width at half maximum (FWHM) in nanometers; in our study, this width is 30 nm.
- Calculating the standard deviation of the Gaussian filter corresponding to the defined FWHM is necessary because the shape of the Gaussian is determined by its standard deviation.
- Calculation of the central wavelength of each Gaussian filter. we use a distance of 3 times the standard deviation of the start wavelength. Then, we move at a calculated interval between filters and end at a distance of 3 times the standard deviation of the end wavelength. Subsequently, we round the values to the nearest integer and sample at the desired spectral interval.
- Creation of Gaussian filters using a Gaussian function. Each filter is calculated based on the similarity between the spectral wavelength and the central wavelength of the filter. The greater the similarity, the higher the filter weight. Filters are normalized to ensure that their sum equals 1.
- The recovery of original image data from 31 bands is followed by filtering using the created Gaussian filters.
- Multiplication of Gaussian filters to the weighted data to perform the 8-band multispectral transformation, selecting the appropriate spectral bands.

The 430, 464, 498, 529, 571, 605, 645, and 680 nm bands used for projection result from optimization work with the genetic algorithm.

In this approach, it is assumed that there is no change in the inclination of the illuminance.

### 3.3. Mosaicking Process to Obtain Sparse Images

**Figure 2:**
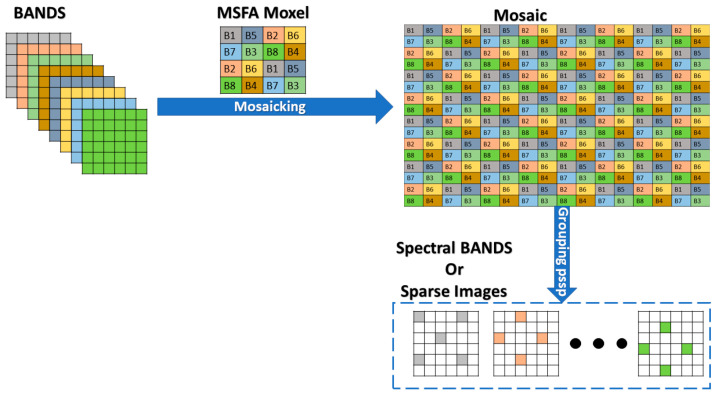
Process of mosaicking and grouping pixels with similar spectral properties.

A mosaic image captured by our camera produces 8 sparse images after grouping pixels with similar spectral properties. Since we will be working with fully defined images, we use our MSFA moxel to generate mosaics from them. Figure 2 illustrates the mosaicking process with our MSFA moxel and the grouping of pixels with similar spectral properties into sparse images.

**Figure 3:**
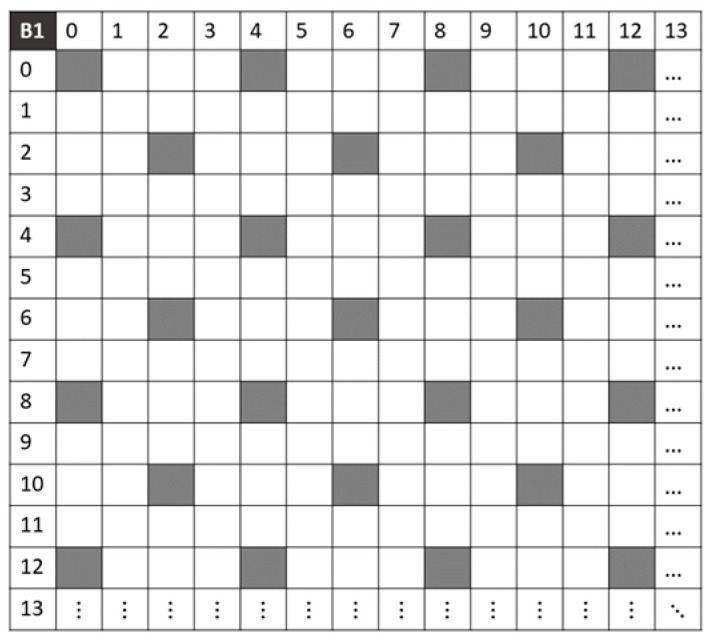
Spatial distribution of pixels in the sparse image of spectral band B1.

Figure 3 shows the spatial distribution of pixels in the sparse images of spectral band B1. The gray areas represent the available pixels, while the white areas represent the null pixels.

Our approach is to reduce the number of null pixels in these sparse images. We expect that reducing the number of null pixels will reduce reconstruction errors during the demosaicing process.

### 3.4. Conceptualization of the Method

Let us define $B=\left\{B_{1},\;B_{2},\;B_{3},\;{\;B}_{4},\;B_{5},\;B_{6},\;B_{7},\;B_{8}\right\}$, the original spectral bands that have the fully defined information (pixels) of the eight bands obtained after projection.

Let us define $I^{MSFA}$ as the mosaic obtained after the first snapshot without sensor displacement. Synthetically, it is obtained using our MSFA moxel on $B_{i}$ bands.

Let us define $I_{k_{j}D_{j}}^{MSFA}$ as the mosaic obtained with camera displacement of *k_j_* pixels, where *k_j_* ∈ {1, …, 3}, along the *D_j_* axes, which can be either horizontal (*H*) or vertical (*V*). Synthetically, these mosaics are obtained by shifting the bands $B_{i}$ of *k_j_* pixels along the *D_j_* axes. This produces bands $B_{ij}^{\prime }$, which are mosaicked with our MSFA moxel.

**Figure 4:**
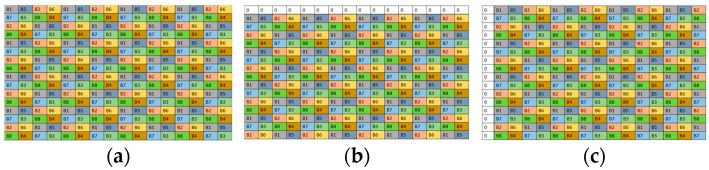
Mosaics obtained before and after camera displacement. (**a**) $I^{MSFA}$ mosaic obtained with main snapshot. (**b**) $I_{1V}^{MSFA}$ mosaic obtained with dispacement of camera on the vertical axis of 1 pixel. (**c**) $I_{1H}^{MSFA}$ mosaic obtained with displacement of camera on the horizontal axis of 1 pixel.

Figure 4 illustrates the different mosaics obtained with a one-pixel camera displacement on the vertical axis (*k*_1_ = 1 and *D*_1_ = *V)* and a one-pixel camera displacement on the horizontal axis (*k*_2_ = 1 and *D*_2_ = *H*).

These mosaic matrices have the following shapes for *k_j_* displacement:

$$\begin{array}{cc} \underset{I^{MSFA}}{\left(\begin{array}{cc} \begin{array}{cc} B_{1}^{\left(0,0\right)} & B_{5}^{\left(0,1\right)}\\ B_{7}^{\left(1,0\right)} & B_{3}^{\left(1,1\right)} \end{array} & \begin{array}{cc} B_{2}^{\left(0,2\right)} & B_{6}^{\left(0,3\right)}\\ B_{8}^{\left(1,2\right)} & B_{4}^{\left(1,3\right)} \end{array}\\ \begin{array}{cc} B_{2}^{\left(2,0\right)} & B_{6}^{\left(2,1\right)}\\ B_{8}^{\left(3,0\right)} & B_{4}^{\left(3,1\right)} \end{array} & \begin{array}{cc} B_{1}^{\left(2,2\right)} & B_{5}^{\left(2,3\right)}\\ B_{7}^{\left(3,2\right)} & B_{3}^{\left(3,3\right)} \end{array}\\ \begin{array}{cc} \vdots & \vdots \end{array} & \begin{array}{cc} \vdots & \vdots \end{array} \end{array}\;\;\;\;\begin{array}{c} \begin{array}{cc} B_{1}^{\left(0,4\right)} & \cdots \\ B_{7}^{\left(1,4\right)} & \cdots \end{array}\\ \begin{array}{cc} B_{2}^{\left(2,4\right)} & \cdots \\ B_{8}^{\left(3,4\right)} & \cdots \end{array}\\ \begin{array}{cc} \vdots & \vdots \end{array} \end{array}\right)} & \underset{I_{k_{1}V}^{MSFA}}{\left(\begin{array}{ccc} \begin{array}{cc} \begin{array}{c} \vdots \\ B_{1}^{\left(k_{1},0\right)} \end{array} & \begin{array}{c} \vdots \\ B_{5}^{\left(k_{1},1\right)} \end{array}\\ B_{7}^{\left(k_{1}+1,0\right)} & B_{3}^{\left(k_{1}+1,1\right)} \end{array} & \begin{array}{cc} \begin{array}{c} \vdots \\ B_{2}^{\left(k_{1},2\right)} \end{array} & \begin{array}{c} \vdots \\ B_{6}^{\left(k_{1},3\right)} \end{array}\\ B_{8}^{\left(k_{1}+1,2\right)} & B_{4}^{\left(k_{1}+1,3\right)} \end{array} & \begin{array}{cc} \begin{array}{c} \vdots \\ B_{1}^{\left(k_{1},4\right)} \end{array} & \begin{array}{c} \cdots \\ \cdots \end{array}\\ B_{7}^{\left(k_{1}+1,4\right)} & \cdots \end{array}\\ \begin{array}{cc} B_{2}^{\left(k_{1}+2,0\right)} & B_{6}^{\left(k_{1}+2,1\right)}\\ \begin{array}{c} B_{8}^{\left(k_{1}+3,0\right)}\\ \vdots \end{array} & \begin{array}{c} B_{4}^{\left(k_{1}+3,1\right)}\\ \vdots \end{array} \end{array} & \begin{array}{cc} B_{1}^{\left(k_{1}+2,2\right)} & B_{5}^{\left(k_{1}+2,3\right)}\\ \begin{array}{c} B_{7}^{\left(k_{1}+3,2\right)}\\ \vdots \end{array} & \begin{array}{c} B_{3}^{\left(k_{1}+3,3\right)}\\ \vdots \end{array} \end{array} & \begin{array}{cc} B_{2}^{\left(k_{1}+2,4\right)} & \cdots \\ B_{8}^{\left(k_{1}+3,4\right)} & \cdots \\ \vdots & \ddots \end{array} \end{array}\right)} \end{array}$$

$$\underset{I_{k_{2}H}^{MSFA}}{\left(\begin{array}{cc} \begin{array}{cc} \cdots & B_{1}^{\left(0,k_{2}\right)}\\ \cdots & B_{7}^{\left(1,k_{2}\right)} \end{array} & \begin{array}{cc} B_{5}^{\left(0,k_{2}+1\right)} & B_{2}^{\left(0,k_{2}+2\right)}\\ B_{3}^{\left(1,k_{2}+1\right)} & B_{8}^{\left(1,k_{2}+2\right)} \end{array}\\ \begin{array}{cc} \cdots & B_{2}^{\left(2,k_{2}\right)}\\ \begin{array}{c} \cdots \\ \ldots \end{array} & \begin{array}{c} B_{8}^{\left(3,k_{2}\right)}\\ \vdots \end{array} \end{array} & \begin{array}{cc} B_{6}^{\left(2,k_{2}+1\right)} & B_{1}^{\left(2,k_{2}+2\right)}\\ \begin{array}{c} B_{4}^{\left(3,k_{2}+1\right)}\\ \vdots \end{array} & \begin{array}{c} B_{7}^{\left(3,k_{2}+2\right)}\\ \vdots \end{array} \end{array} \end{array}\;\;\;\;\begin{array}{c} \begin{array}{cc} B_{6}^{\left(0,k_{2}+3\right)} & \begin{array}{cc} B_{1}^{\left(0,k_{2}+4\right)} & \cdots \end{array}\\ B_{4}^{\left(1,k_{2}+3\right)} & \begin{array}{cc} B_{7}^{\left(1,k_{2}+4\right)} & \cdots \end{array} \end{array}\\ \begin{array}{cc} B_{5}^{\left(2,k_{2}+3\right)} & \begin{array}{cc} B_{2}^{\left(2,k_{2}+4\right)} & \cdots \end{array}\\ \begin{array}{c} B_{3}^{\left(3,k_{2}+3\right)}\\ \vdots \end{array} & \begin{array}{cc} \begin{array}{c} B_{8}^{\left(3,k_{2}+4\right)}\\ \vdots \end{array} & \cdots \end{array} \end{array} \end{array}\right)}$$

The process of grouping pixels with similar spectral properties involves separating a mosaic image into different spectral bands using a binary mask formulated as follows:

(1) $$m_{B}^{i}\left(p\right)=\left\{\begin{array}{ll} 1, & p\;\in B_{i}\\ 0, & otherwise \end{array}\right.$$

For each mosaic, we obtain a set of sparse images, ${\tilde{I}}^{i}$, by applying the following formula on them:

(2) $${\tilde{I}}^{i}=I^{MSFA}⊙m_{B}^{i}$$

For any camera displacement, we obtain the mosaics $I_{k_{j}D_{j}}^{i}\;with\;i\in \left\{1,\ldots ,\;8\right\},\;k_{j}\in \left\{1,\ldots ,\;3\right\},\;and\;D_{j}\;\epsilon \;\left\{H,\;V\right\},$ where *i* represents the index of a band of the MSFA moxel and *k_j_* represents the displacement scalars along the horizontal (*H*) and vertical (*V*) axes.

**Figure 5:**
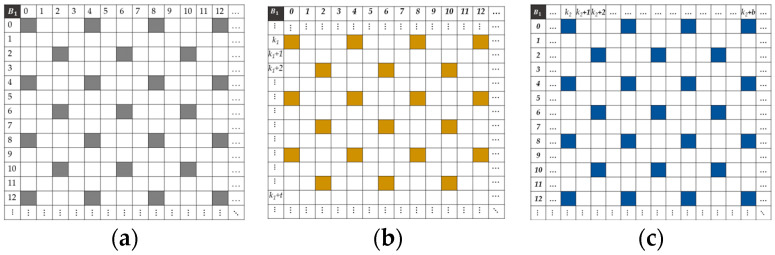
Spatial distribution of pixels in the sparse image of the spectral band B_1_. (**a**) ${\tilde{I}}^{1}$ is the sparse image of spectral band B_1_. (**b**) ${\tilde{I}}_{k_{1}V}^{1}$ is the sparse image of spectral band B_11_ with the camera displacement on the vertical axis of *k*_1_ pixels. (**c**) ${\tilde{I}}_{k_{2}H}^{1}$ is the sparse image of spectral band B_12_ with the camera displacement on the horizontal axis of *k*_2_ pixels.

Figure 5 shows the density of pixels that are spectrally similar in band B1 of the mosaics $I^{MSFA},\;I_{k_{1}H}^{MSFA},$ and $I_{k_{2}V}^{MSFA}$. The gray areas represent the available pixels in the sparse image ${\tilde{I}}^{1}$ of the band *B*_1_; the yellow areas represent those available in the sparse image ${\tilde{I}}_{k_{1}V}^{1}$ of the band $B_{11}^{\prime }$ due to the camera’s displacement on the vertical axis of *k*_1_ pixels; and the blue areas represent the pixels available in the sparse image ${\tilde{I}}_{k_{2}H}^{1}$ of the band $B_{12}^{\prime }$ due to the camera’s displacement on the vertical axis of *k*_2_ pixels.

The positions of the non-null pixels vary in each sparse image, and these pixels have the same spectral properties. Therefore, the sparse images can be combined (composition method), i.e., added together, to increase the number of non-null pixels and reduce the number of null pixels. The pixels are redistributed according to the camera displacement combinations.

### 3.5. Sparse Image Composition

**Figure 6:**
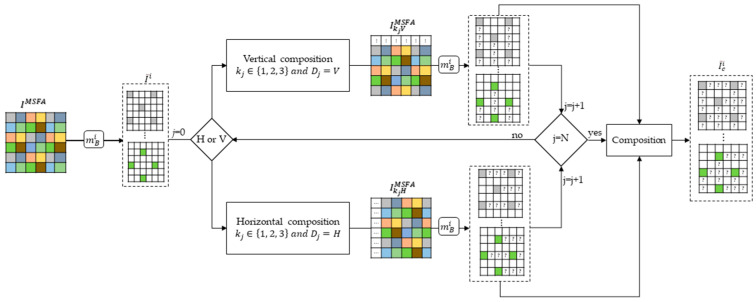
Architecture of our composition method.

The sparse image composition method is performed in 3 steps, as shown in Figure 6. The first step is to take an initial snapshot of a scene. This snapshot provides a mosaic image $I^{MSFA}$, which is decomposed into sparse images ${\tilde{I}}^{i}$ using Formula (2). Then we set the number N of compositions we want to make by specifying the displacement scalars *k_j_*, and the axes *D_j_*. Finally, we obtain composite sparse images ${\tilde{I}}_{c}^{i}$, which contain more available pixels. The symbol “?” in the composite sparse images ${\tilde{I}}_{c}^{i}$, indicates the areas where new pixels can appear depending on the displacement combination. This composition method reduces the distance between two non-null pixels and is limited to three compositions. Beyond three compositions, implementing such a method can be very time consuming.

#### 3.5.1. Case of the Composition of Two Sparse Images

For two bands, we obtain six possible compositions for the different values of the displacement scalar on the two axes *H* and *V*. The following algorithm shows how the composition of two bands is achieved:

- The camera takes a first snapshot from which we obtain a mosaic $I^{MSFA}$;
- The camera moves *k* pixel(s) on the *D* axis and takes a second snapshot, from which a second mosaic $I_{kD}^{MSFA}$ is obtained;
- The separation into sparse image is performed on the mosaics $I^{MSFA}$ and $I_{kD}^{MSFA}$ with Formula (2), resulting in sparse images ${\tilde{I}}^{i}$ and ${\tilde{I}}_{kD}^{i}$;
- The addition of the two sparse images is performed, such that ${\tilde{I}}_{c}^{i}={\tilde{I}}^{i}+{\tilde{I}}_{kD}^{i}$.

**Figure 7:**
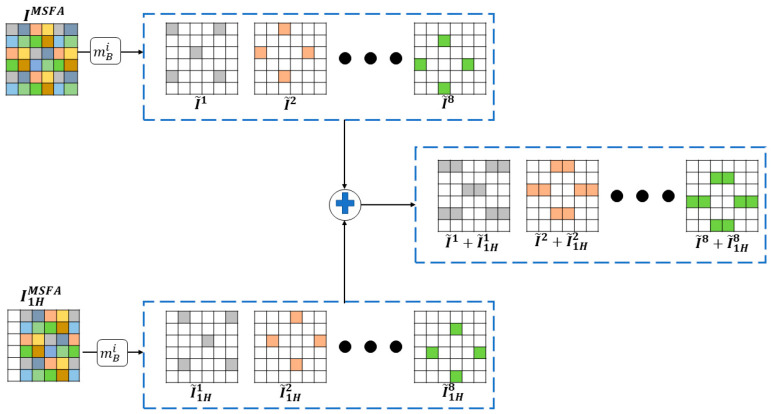
Composition scheme of the two sparse images of the $I^{MSFA}$ and $I_{1H}^{MSFA}$ mosaics.

Figure 7 shows a composition of bands from the $I^{MSFA}$ and $I_{1H}^{MSFA}$ mosaics. The eight sparse images have globally the same pixel distributions, which vary according to the parameters *k_j_* and *D_j_*. Thus, for a given camera displacement, the pixel distribution in the composite sparse image ${\tilde{I}}_{c}^{i}$ is the same, which justifies that we comment only on spectral band B1 of each composition.

**Figure 8:**
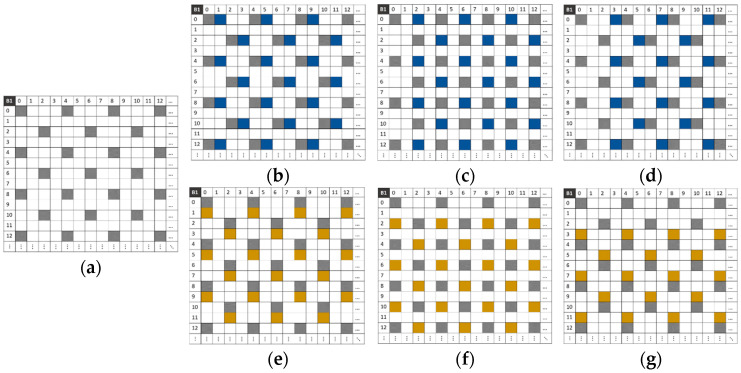
Pixel distribution in the composite sparse image ${\tilde{I}}_{c}^{1}$ for each camera displacement. (**a**) ${\tilde{I}}_{c}^{i}$ without displacement. (**b**–**d**) ${\tilde{I}}_{c}^{i}$ with displacements of 1, 2, and 3 pixels on the horizontal axis, respectively. (**e**–**g**) ${\tilde{I}}_{c}^{i}$ with displacements of 1, 2, and 3 pixels on the vertical axis, respectively.

Figure 8 shows the six possible compositions of band B1 with different values of the displacement scalar *k_j_* on the *V* and *H* axes. The new composition allows for more pixels and better redistribution to minimize the non-null pixel distance in the composite sparse images ${\tilde{I}}_{c}^{i}$.

#### 3.5.2. Cases of Sparse Images Greater Than Two

For more than two bands, we obtain more than 30 possible compositions for the different values of the displacement scalars *k_j_* on the axes *D_j_*. The following algorithm shows how the composition of *N* bands is achieved where 2<N≤4:

1. The camera takes a first snapshot from which a mosaic $I^{MSFA}$ is obtained.
2. The separation into sparse images is performed on the mosaics $I^{MSFA}$ using Formula (2), resulting in the sparse images ${\tilde{I}}^{i}$.
3. The initialization step sets the values of ${\tilde{I}}_{c}^{i}$ to ${\tilde{I}}^{i}$ and *j* to 1.
4. As long as *j* ≤ *N*:
  - The camera moves along the *D_j_* axis by *k_j_* pixels from its position (0, 0) and takes a snapshot, and a new mosaic $I_{k_{j}D_{j}}^{i}$ is obtained.
  - The new mosaic $I_{k_{j}D_{j}}^{i}$ is decomposed using Formula (2), resulting in sparse images ${\tilde{I}}_{k_{j}D_{j}}^{i}$.
  - The above sparse image is added to the previous sparse image ${\tilde{I}}_{c}^{i}={\tilde{I}}_{c}^{i}+{\tilde{I}}_{k_{j}D_{j}}^{i}$, and the value of *j* is incremented.
5. In the end, we get composite sparse images ${\tilde{I}}_{c}^{i}$.

**Figure 9:**
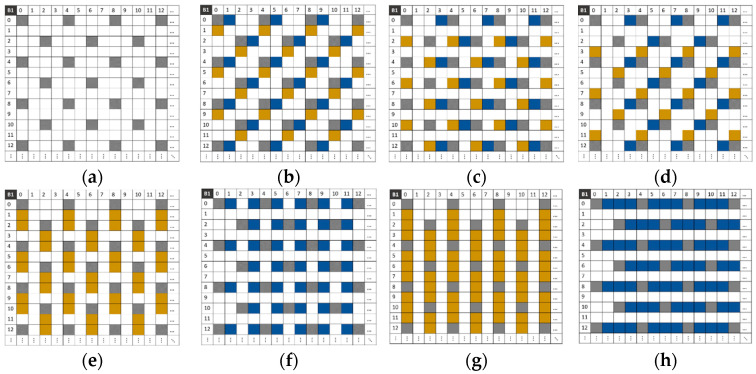
Pixel distribution in the composite sparse image ${\tilde{I}}_{c}^{1}$ for each camera displacement. (**a**) ${\tilde{I}}_{c}^{i}$ without displacement. (**b**) ${\tilde{I}}_{c}^{i}$ with two displacements of 1 pixel on both the *H*- and *V*-axis. (**c**) ${\tilde{I}}_{c}^{i}$ with two displacements of 3 pixels on the *H*-axis and 2 pixels on the *V*-axis. (**d**) ${\tilde{I}}_{c}^{i}$ with two displacements of 3 pixels each on the *H*- and *V*-axis. (**e**) ${\tilde{I}}_{c}^{i}$ with two displacements of 1 and 2 pixels on the same *V*-axis. (**f**) ${\tilde{I}}_{c}^{i}$ with two displacements of 1 and 3 pixels on the same *H*-axis. (**g**) ${\tilde{I}}_{c}^{i}$ with three displacements of 1, 2, and 3 pixels on the same *V*-axis. (**h**) ${\tilde{I}}_{c}^{i}$ with three displacements of 1, 2, and 3 pixels on the same *H*-axis.

Figure 9 illustrates the spatial distribution of pixels of certain three- and four-band compositions. The blue area represents the *H*-axis displacement and the yellow area represents the *V*-axis displacement.

The composition method redistributes pixels to provide more information and reduce the number of pixels to interpolate. It is important to note that with our MSFA moxel it is not possible to achieve a three-band composition with a displacement of two pixels on both the horizontal and vertical axes (*k*_1_ = *k*_2_ = 2 on the *H* and *V* axes). This would cause a problem with overlapping pixels at certain positions of the composite sparse image.

### 3.6. Bilinear Interpolation

To generate a fully defined image, we use the bilinear interpolation on the sparse images to deduce the null pixels according to the following Algorithm 1:

**Algorithm 1:** Bilinear interpolation.**Input:** sparse_image, method**Output:** InterpIMG
**BEGIN**
 Width = sparse_image.width Height = sparse_image.height XI = value grid going from 1 to height + 1 YI = value grid going from 1 to width + 1 Ind = coordinates of data to interpolate Z = values of non-null indices InterpIMG = **grid_interpolation**(Ind,Z,(XI, YI), fill_value = 2.2 × 10^−16^)
**END**


We set the fill value to 2.2 × 10^−16^ to avoid the zero-value. This would avoid having *nan* values in our interpolated matrix. The grid_interpolation function is given in the following Algorithm 2.

**Algorithm 2:** grid_interpolation.**Input:** points, values, grid, method, fill_value  // points: The coordinates of the data to interpolate  // values: The corresponding values at the data points  // grid: The grid on which to interpolate the data  // fill_value: the value to use for points outside the input grid**Output:** InterpIMG
**BEGIN**
  **For** each point (x, y) in grid:    **If** (x, y) is outside of the input points:      **Assign** fill_value to InterpIMG(x, y)   **Else:**       **Find** the k (2 ≤ k ≤ 4) nearest data points within a rectangular grid, with 2 along each axis       **Calculate** the weights for interpolation based on distance       **Interpolate** the value at (x, y) using the input values in points and interpolation weights      **Assign** the new value to InterpIMG(x, y)   **End If**  **End For**
**End If**

**END**


### 3.7. The General Architecture of Our Method

**Figure 10:**
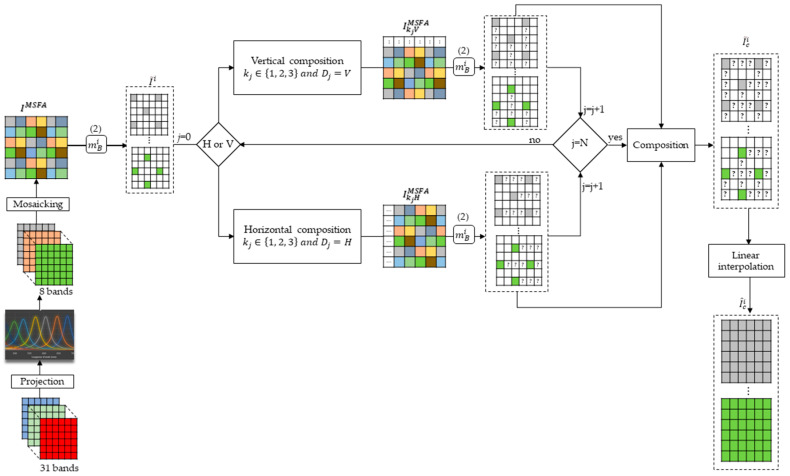
General architecture of our projection, composition, and interpolation method.

The architecture in Figure 10 shows the general flow of our method. We start by projecting the images of TT-31 into 8 bands. Then, we create a mosaic from these bands, which represents the first snapshot of the sensor. The mosaic is decomposed into 8 sparse images that go through a composition method that depends on the displacement of the sensor horizontally or vertically. Each displacement provides a mosaic that is decomposed into 8 sparse images, which are added together with the previous 8 sparse images to form the new composite sparse images. The composition process is repeated until the stop condition is reached. The final composite sparse images are demosaiced using a bilinear method to obtain the fully reconstructed images.

## 4. Experiments and Results

Our experiments aim to demonstrate how reducing the number of null pixels in sparse images can improve the quality of the spatial resolution obtained after interpolation. To achieve this, we will compare the fully reconstructed images of sparse images and composite sparse images. We will use the TokyoTech datasets TT-31 projected on the response of our MSFA filter, on which we will perform qualitative and quantitative analyses to determine the impact of this approach.

### 4.1. Metric

To evaluate our results, we use four quantitative metrics, namely:

- PSNR (Peak Signal to Noise Ratio) [29]: PSNR is a widely used metric to assess the quality of a reconstructed or compressed image compared to the original image. This metric measures the ratio between the maximum power of the signal (which is called the peak signal) and the power of the noise that degrades the quality of the image representation (also known as the corrupting noise). Higher PSNR values indicate better image quality because they represent a higher ratio of signal power to noise power.
  (3) $$PSNR=10\cdot \log_{10}\left(\frac{{\left(2^{n}-1\right)}^{2}}{\frac{1}{n}\sum_{i=1}^{n}{\left(I^{i}-{\hat{I}}^{i}\right)}^{2}}\right)$$
  where *n* is the number of spectral bands in the MSFA moxel.
- SAM (Spectral Angle Metric) [30] calculates the angle between two spectral vectors in a high-dimensional space. Each spectral vector represents the spectral reflectance or irradiance of a pixel over several spectral bands.
  (4) $$SAM=\cos^{-1}\left(\frac{\sum_{i=1}^{n}\left(I^{i}⊙{\hat{I}}^{i}\right)}{\sqrt{\sum_{i=1}^{n}\left({I^{2}}^{i}⊙{{\hat{I}}^{2}}^{i}\right)}}\right)$$
  The smaller the angle between two spectral vectors, the more similar the spectra are considered to be.
- SSIM (Structural Similarity Index Measure) [31]: SSIM is a method used to measure the similarity between two images. This technique compares the structural information, luminance, and contrast of the two images, taking into account the characteristics of the human visual system. Compared to simpler metrics such as Mean Square Error (MSE) or PSNR, SSIM provides a more comprehensive assessment of image similarity by considering perceptual factors. The SSIM value ranges from 0 to 1, where
  ∘ 1 indicates perfect similarity between images.
  ∘ 0 indicates no similarity between images.
  We use the structural_similarity function of the python skimage.metrics module to compute this metric.
- RMSE (Root Mean Square Error) [32]: RMSE is a commonly used metric to evaluate the accuracy of predictions by measuring the average size of the errors between the predicted and actual values in a given set of predictions. The metric is expressed in the same unit as the target value. For example, if the target value is to predict a certain value, and we obtain an RMSE of 10, this indicates that the predicted value varies on average by ±10 from the actual value. The formula for calculating the RMSE is as follows:
  (5) $$RMSE=\sqrt{\frac{\sum_{i=1}^{n}{\left(I^{i}-{\hat{I}}^{i}\right)}^{2}}{n}}$$

### 4.2. Quantitative Evaluation

We have conducted tests on 20 images from the TokyoTech database, and the quantitative results of the PSNR, SAM, SSIM, and RMSE metrics are presented in Table 1, Table 2, Table 3 and Table 4. The header “Snapshots” indicates the number of snapshots taken by the camera. For example, taking *p* snapshots, where *p* ∈ {2, …, *n*}, means taking one snapshot without displacement of the camera and taking *p* − 1 other snapshots by displacements of the camera along the specified axes. The “Displacements” header of the tables specifies the different configurations of sparse image compositions, identified by the letter compositions, where

- ‘*a*’ corresponds to a snapshot without displacement
- ‘*b*’ corresponds to a snapshot after a displacement of 1 pixel on the *H*-axis
- ‘*c*’ corresponds to a snapshot after a displacement of 1 pixel on the *V*-axis
- ‘*d*’ corresponds to a snapshot after a displacement of 2 pixels on the *H*-axis
- ‘*e*’ corresponds to a snapshot after a displacement of 2 pixels on the *V*-axis
- ‘*f*’ corresponds to a snapshot after a displacement of 3 pixels on the *H*-axis
- ‘*g*’ corresponds to a snapshot after a displacement of 3 pixels on the *V*-axis

The ‘*abc*’ displacements represent three different snapshots taken by a camera. The first snapshot is taken without any displacement, the second snapshot is taken after a horizontal displacement of one pixel, and the third snapshot is taken after a vertical displacement of one pixel. The values in each cell of the table represent the average of the eight spectral bands. Using this quantitative evaluation method, we can compare the values obtained from composite sparse images with the values obtained from sparse images without any composition. Note that the tables do not cover all possible combinations of camera displacements but only a selection. The results indicate that images reconstructed from composed bands are of higher quality than those reconstructed without band composition. However, the quality of the reconstructed image depends not only on the specific composition used but also on the individual image.

### 4.3. Qualitative Assessment

**Figure 11:**
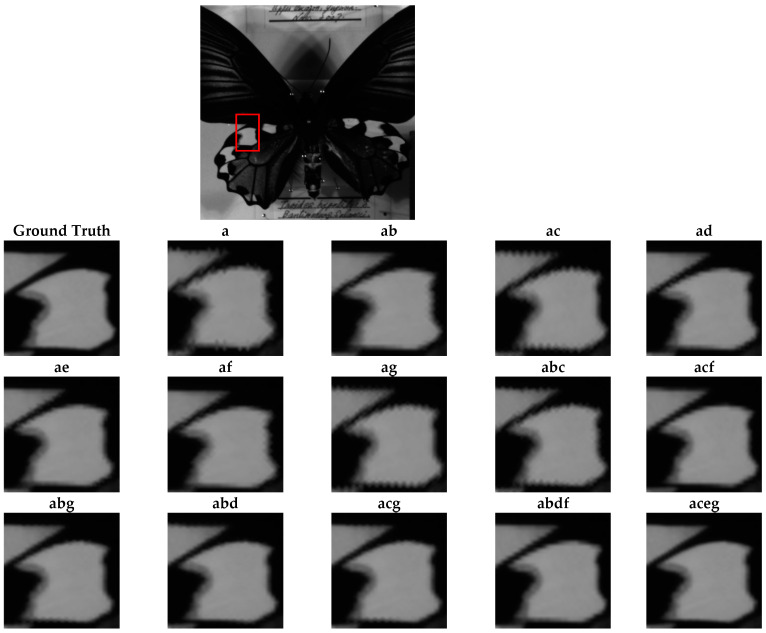
Qualitative assessment of the Butterfly8 image according to different compositions.

**Figure 12:**
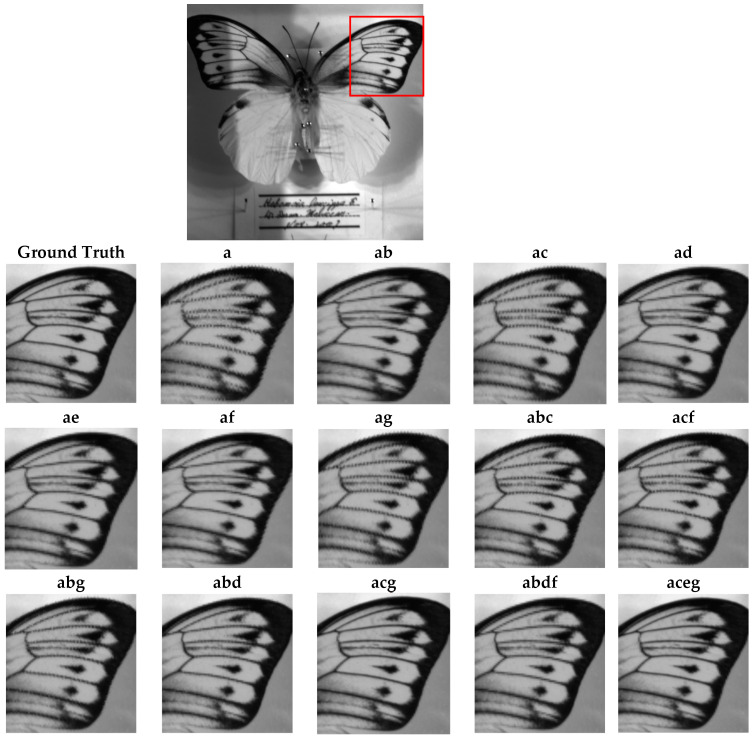
Qualitative assessment of the Butterfly image according to different compositions.

**Figure 13:**
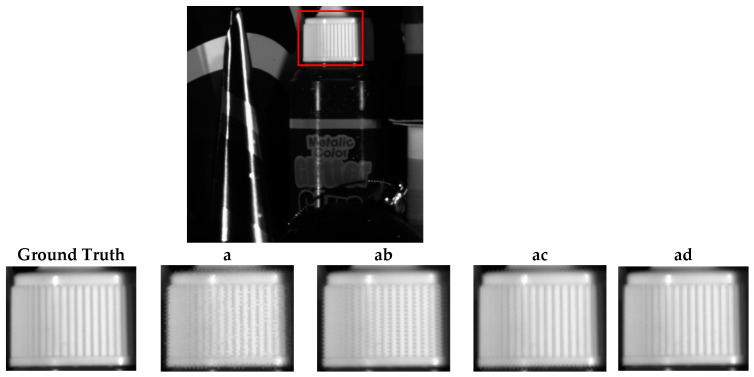

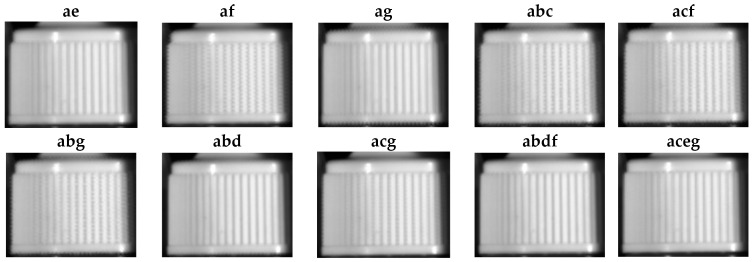
Qualitative assessment of the Party image according to different compositions.

Figure 11, Figure 12 and Figure 13 show the reconstructions of fine details in images containing abrupt transitions, non-homogeneous areas (Figure 11 and Figure 12), and textured regions (Figure 13). We will compare the quality of images reconstructed from sparse and composite sparse images by selecting and zooming in on a 60 × 60 pixel area from “Butterfly8”, a 155 × 165 pixel area from “Butterfly”, and a 130 × 104 pixel area from “Party”. The selected areas are indicated by red boxes in the original images. The quality of the reconstructions improves from two snapshots to four. We also note a significant correlation between the spatial distribution of pixels in the sparse image and the reconstruction results.

## 5. Discussion

The study’s results show a direct correlation between the number of compositions and the spatial resolution of the reconstructed image, especially when reconstructing abrupt transitions, non-homogeneous areas, and textured regions. The more compositions performed, the better the reduction of the distance between the non-null pixels of the sparse images, leading to a better spatial resolution after demosaicing. For each level of composition, there are differences in the qualitative and quantitative results depending on the values of the displacement scalars.

Several observations can be made about compositions involving two bands where only one camera displacement is required. For certain images in Figure 11 and Figure 12, there is a preference for horizontal shifts, while for others in Figure 13, there is a preference for vertical shifts. Depending on the type of image, there is a clear improvement in abrupt transitions, non-homogeneous areas, and textured regions. Two-pixel displacements significantly improve local structures such as edges, textures, and patterns compared to the image obtained without band compositing. This improvement is manifested in higher SSIM values (Table 3), lower spectral similarity angle according to SAM (Table 2), and lower reconstruction error according to RMSE (Table 4). However, reconstruction with less noise is observed with 1-pixel or 3-pixel displacements, as shown by PSNR (Table 1). The study highlights a significant correlation between the spatial distribution of the pixels in the sparse images and the quantitative and qualitative results after reconstruction. Indeed, the displacement of 2 pixels better reduces the distance between two non-null pixels of the sparse images, leading to less overlapping in abrupt transitions and improved visual restitution, as shown by the displacement (ad, ae) in Figure 11, Figure 12 and Figure 13. In conclusion, vertical shifts, especially those of 2 pixels, offer a good compromise between improving local structures and reducing noise in the reconstructed images. The study highlights the importance of considering the spatial distribution of pixels when planning camera shifts for optimal reconstruction.

In compositions with three bands and two camera displacements, there are 14 possible combinations of displacements on the horizontal and vertical axes. According to PSNR, 1-pixel or 3-pixel displacements on both axes result in a less noisy reconstruction. SSIM shows that the structural reconstruction is almost equivalent in most cases. Moving along the same axis results in higher spectral similarity and fewer reconstruction errors, as indicated by SAM and RMSE. Visual results show increased sharpness for displacements on the same axis, but decreased sharpness for displacements of 1 pixel on both axes and 3 pixels on both axes. In conclusion, displacements on the same axis provide an optimal compromise between the structural and spectral quality of the reconstruction. At the same time, other configurations offer specific advantages and disadvantages in terms of noise reduction and visual sharpness.

The visual results obtained are very close to the reference image for four-band compositions with three camera displacements, with 10 possible combinations. This suggests a satisfactory ability to reconstruct images with a high level of visual fidelity, although the metrics show less good results than in the case of the three-band composition. However, the implementation of this type of shift is not directly feasible in a real-time acquisition system due to the increased complexity of the camera shift. Therefore, the use of this type of composition is not necessary in real-time acquisition systems. Nevertheless, the displacements of this type of composition on the same axis show excellent visual results. This observation suggests that a limited camera displacement for this type of composition may be sufficient to significantly improve the quality of reconstructed images without requiring the excessive complexity of a bi-axial composition. In conclusion, four-band compositions can produce satisfactory visual results, but their practical implementation in a real-time acquisition system is limited due to their displacement complexity. However, simpler strategies, such as moving along the same axis, can provide significant improvements while reducing the difficulty of operational feasibility.

**Figure 14:**
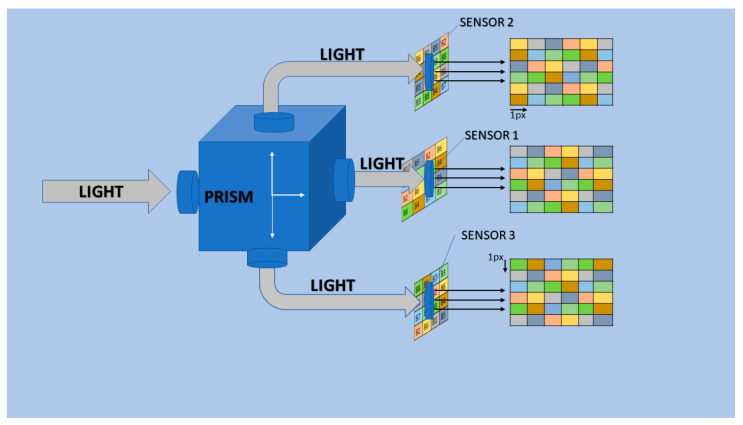
Tri-CCD system diffuses light to three sensors.

In practice, the implementation of our method is possible, in particular, by using a tri-CCD system to capture and restore a motion scene of objects [33]. This acquisition system has a beam splitter to split the light into two other axes. The prism redirects light to three sensors that capture a mosaic of the same scene with different observations, providing three mosaics of the same scene with different spatial information distributions. For static objects, a micron-precision camera translation system would be required to capture and restore the fully defined image. Figure 14 illustrates the operation of a tri-CCD system where each sensor is equipped with an MSFA.

The first MSFA filter is mounted on top of sensor 1 to obtain a mosaic with no information shift. The second MSFA filter is mounted on top of sensor 2 to obtain a mosaic with information shifted by 1 pixel on the horizontal axis. Finally, a third MSFA filter is mounted on top of sensor 3 to obtain a mosaic with information shifted by 1 pixel on the vertical axis.

## 6. Conclusions

This paper presents a first prototype simulation approach to improve the spatial resolution of multispectral images acquired by our MSFA single-shot camera, with particular emphasis on reproducing fine details, abrupt transitions, and textured regions. Our approach proposes a method of camera displacement along horizontal and/or vertical axes to capture multiple snapshots, thus generating different mosaics for the same observed scene. We then proceed to assemble the spectrally similar pixels of these mosaics to increase the number of non-null pixels in the sparse images. The results of our experiments carried out on TT-31 images projected on the response of our MSFA filter show a qualitative and quantitative improvement in the reconstruction based on composite sparse images, with better results validated by the PSNR, SAM, SSIM, and RMSE metrics. The next step will be implementing our multi-shot prototype on our camera by installing a micron-precision camera motion device. This will allow us to perform experiments on real images and propose a new demosaicing method based on composite sparse bands.

## Figures and Tables

**Table 1:** PSNR comparison between single and multiple snapshots.

Snapshots	1	2						3						4	
Displacements	a	ab	ac	ad	ae	af	ag	abc	acf	abg	afg	abd	acg	abdf	aceg
Butterfly	25.46	26.07	25.94	25.56	25.55	26.07	25.94	26.53	26.44	26.47	26.53	26.47	26.44	26.06	25.89
Butterfly2	24.19	24.49	24.66	24.35	24.35	24.49	24.66	24.90	24.62	24.70	24.90	24.71	24.71	24.44	24.59
Butterfly3	28.31	28.56	28.91	28.39	28.39	28.56	28.90	29.10	28.95	29.03	29.10	29.01	28.99	28.51	28.83
Butterfly4	27.86	28.13	28.49	27.94	27.93	28.13	28.49	28.70	28.59	28.62	28.70	28.60	28.53	28.08	28.34
Butterfly5	28.38	29.06	28.56	28.46	28.46	29.07	28.56	29.17	29.09	29.09	29.17	29.06	29.01	29.01	28.44
Butterfly6	25.84	26.36	26.20	25.97	25.96	26.36	26.20	26.66	26.54	26.54	26.65	26.54	26.53	26.32	26.14
Butterfly7	27.54	27.88	28.20	27.64	27.63	27.88	28.20	28.51	28.34	28.43	28.51	28.43	28.40	27.85	28.15
Butterfly8	27.97	28.08	28.64	28.04	28.04	28.09	28.64	28.69	28.40	28.56	28.68	28.51	28.48	28.00	28.49
Colorchart	27.32	27.68	27.90	27.40	27.40	27.68	27.90	28.20	28.20	28.20	28.20	28.18	28.14	27.66	27.83
CD	33.87	34.12	34.20	34.01	34.02	34.12	34.20	34.39	34.31	34.29	34.38	34.31	34.32	34.13	34.19
Cloth2	27.48	28.12	27.73	27.69	27.68	28.11	27.73	28.37	28.13	28.15	28.35	28.19	28.24	28.11	27.75
Cloth3	29.86	30.15	30.11	30.09	30.08	30.15	30.12	30.27	30.02	30.05	30.27	29.87	30.05	29.89	30.05
Cloth6	29.19	29.86	29.43	29.35	29.34	29.86	29.43	30.01	29.82	29.84	29.99	29.77	29.80	29.71	29.35
Flower	29.17	29.53	29.61	29.28	29.28	29.53	29.61	29.89	29.69	29.80	29.89	29.76	29.77	29.46	29.54
Flower2	28.15	28.83	28.55	28.23	28.23	28.84	28.55	29.23	29.13	29.14	29.23	29.13	29.12	28.80	28.50
Flower3	30.91	31.31	31.22	31.03	31.03	31.30	31.22	31.55	31.41	31.45	31.54	31.45	31.45	31.27	31.18
Party	25.97	26.42	26.17	26.10	26.09	26.41	26.16	26.47	26.38	26.39	26.45	26.24	26.32	26.22	26.07
Tape	26.35	26.59	27.17	26.56	26.56	26.59	27.17	27.34	27.15	27.17	27.33	27.13	27.24	26.49	27.24
Tape2	25.25	25.68	25.84	25.55	25.55	25.68	25.84	26.15	25.88	25.99	26.13	26.11	26.07	25.75	25.88
Tshirts2	23.42	23.80	23.72	23.59	23.59	23.81	23.73	23.99	23.71	23.74	23.98	23.53	23.71	23.47	23.64
**Average**	27.62	28.04	28.06	27.76	27.76	28.04	28.06	**28.41**	28.24	28.28	28.40	28.25	28.27	27.96	28.00

**Table 2:** SAM comparison between single and multiple snapshots.

Snapshots	1	2						3						4	
Displacements	a	ab	ac	ad	ae	af	ag	abc	acf	abg	afg	abd	acg	abdf	aceg
Butterfly	1.98	1.55	1.61	1.50	1.50	1.55	1.61	1.42	1.48	1.46	1.42	1.14	1.01	1.23	1.10
Butterfly2	4.29	3.61	3.63	3.27	3.27	3.60	3.63	3.29	3.43	3.42	3.29	2.57	2.58	2.66	2.68
Butterfly3	4.87	4.37	4.38	4.27	4.27	4.37	4.38	4.23	4.34	4.32	4.22	3.98	3.98	4.03	4.05
Butterfly4	3.76	3.36	3.41	3.33	3.33	3.36	3.41	3.26	3.37	3.36	3.25	3.12	3.08	3.15	3.14
Butterfly5	3.23	2.70	2.71	2.56	2.56	2.70	2.72	2.49	2.61	2.60	2.50	2.10	2.08	2.18	2.17
Butterfly6	2.70	2.33	2.35	2.27	2.27	2.32	2.35	2.18	2.27	2.25	2.18	1.99	1.96	2.06	2.03
Butterfly7	3.96	3.36	3.39	3.31	3.30	3.36	3.39	3.30	3.41	3.39	3.29	3.15	3.08	3.19	3.11
Butterfly8	3.53	3.25	3.13	2.79	2.79	3.26	3.12	2.85	3.03	3.01	2.85	2.10	2.46	2.16	2.57
Colorchart	5.16	4.67	4.30	4.16	4.17	4.67	4.30	4.18	4.26	4.25	4.18	2.96	3.65	3.04	3.76
CD	3.65	3.40	3.09	3.06	3.06	3.40	3.09	2.99	3.12	3.10	2.99	2.24	2.75	2.32	2.85
Cloth2	3.73	3.10	3.24	2.99	2.99	3.10	3.24	2.99	3.04	3.03	2.99	2.60	2.50	2.66	2.58
Cloth3	4.40	3.57	3.59	3.31	3.30	3.57	3.59	3.39	3.40	3.39	3.39	2.57	2.51	2.64	2.60
Cloth6	4.86	4.00	3.98	3.67	3.68	4.00	3.98	3.70	3.88	3.86	3.71	2.86	2.92	2.93	3.01
Flower	3.21	2.81	2.85	2.77	2.77	2.81	2.84	2.69	2.76	2.75	2.69	2.50	2.53	2.54	2.63
Flower2	3.25	2.85	2.88	2.79	2.79	2.85	2.88	2.73	2.82	2.80	2.72	2.53	2.53	2.57	2.61
Flower3	3.74	3.27	3.37	3.26	3.26	3.27	3.37	3.17	3.26	3.25	3.16	3.01	2.99	3.05	3.10
Party	4.25	3.58	3.34	3.12	3.13	3.58	3.34	3.10	3.28	3.27	3.10	1.95	2.44	2.03	2.55
Tape	2.26	1.97	1.99	1.96	1.96	1.97	1.99	1.82	1.92	1.90	1.83	1.70	1.70	1.78	1.78
Tape2	5.07	4.40	4.44	4.30	4.30	4.40	4.44	4.26	4.41	4.39	4.25	4.00	3.97	4.05	4.03
Tshirts2	5.43	4.50	4.36	3.99	3.99	4.50	4.37	4.05	4.16	4.14	4.05	2.76	3.08	2.84	3.17
**Average**	3.87	3.33	3.30	3.13	3.13	3.33	3.30	3.11	3.21	3.20	3.10	**2.59**	2.69	2.65	2.78

**Table 3:** SSIM comparison between single and multiple snapshots.

Snapshots	1	2						3						4	
Displacements	a	ab	ac	ad	ae	af	ag	abc	acf	abg	afg	abd	acg	abdf	aceg
Butterfly	0.692	0.717	0.719	0.725	0.725	0.717	0.719	0.729	0.710	0.711	0.729	0.722	0.722	0.723	0.723
Butterfly2	0.659	0.686	0.686	0.695	0.695	0.686	0.686	0.700	0.677	0.677	0.699	0.688	0.692	0.690	0.695
Butterfly3	0.674	0.692	0.691	0.694	0.694	0.692	0.691	0.696	0.680	0.680	0.697	0.683	0.682	0.686	0.684
Butterfly4	0.677	0.697	0.697	0.701	0.701	0.697	0.698	0.704	0.685	0.686	0.705	0.690	0.688	0.693	0.690
Butterfly5	0.682	0.710	0.710	0.718	0.718	0.709	0.710	0.723	0.703	0.703	0.723	0.717	0.719	0.717	0.720
Butterfly6	0.686	0.707	0.707	0.711	0.711	0.706	0.707	0.715	0.694	0.694	0.715	0.699	0.699	0.702	0.702
Butterfly7	0.638	0.660	0.658	0.664	0.663	0.660	0.658	0.667	0.647	0.647	0.668	0.653	0.653	0.655	0.656
Butterfly8	0.636	0.658	0.659	0.662	0.662	0.658	0.659	0.663	0.651	0.650	0.663	0.656	0.653	0.659	0.654
Colorchart	0.628	0.656	0.649	0.658	0.659	0.655	0.649	0.660	0.646	0.646	0.660	0.651	0.655	0.653	0.658
CD	0.547	0.597	0.607	0.623	0.622	0.597	0.607	0.631	0.598	0.600	0.629	0.634	0.639	0.632	0.638
Cloth2	0.567	0.606	0.606	0.619	0.619	0.606	0.606	0.624	0.598	0.598	0.623	0.619	0.618	0.621	0.618
Cloth3	0.620	0.650	0.648	0.657	0.657	0.649	0.648	0.661	0.639	0.639	0.660	0.651	0.650	0.654	0.653
Cloth6	0.671	0.694	0.694	0.699	0.699	0.694	0.694	0.702	0.687	0.687	0.702	0.694	0.694	0.696	0.696
Flower	0.671	0.701	0.704	0.712	0.712	0.701	0.704	0.717	0.697	0.698	0.716	0.716	0.714	0.717	0.715
Flower2	0.759	0.773	0.775	0.777	0.777	0.773	0.775	0.779	0.768	0.769	0.779	0.772	0.772	0.773	0.773
Flower3	0.732	0.752	0.753	0.757	0.757	0.752	0.752	0.760	0.746	0.746	0.760	0.753	0.753	0.755	0.754
Party	0.733	0.751	0.750	0.754	0.754	0.751	0.750	0.757	0.744	0.744	0.757	0.749	0.748	0.751	0.749
Tape	0.662	0.679	0.680	0.681	0.681	0.679	0.680	0.683	0.667	0.667	0.683	0.667	0.666	0.670	0.669
Tape2	0.612	0.637	0.636	0.644	0.644	0.637	0.636	0.649	0.620	0.620	0.650	0.627	0.635	0.629	0.639
Tshirts2	0.613	0.660	0.658	0.675	0.675	0.660	0.658	0.681	0.654	0.653	0.680	0.682	0.680	0.682	0.680
**Average**	0.658	0.684	0.684	0.691	0.691	0.684	0.684	**0.695**	0.676	0.676	0.695	0.686	0.687	0.688	0.688

**Table 4:** RMSE comparison between single and multiple snapshots.

Snapshots	1	2						3						4	
Displacements	a	ab	ac	ad	ae	af	ag	abc	acf	abg	afg	abd	acg	abdf	aceg
Butterfly	5.54	5.13	5.16	5.25	5.25	5.13	5.17	4.91	5.03	5.01	4.92	4.89	4.88	5.03	5.06
Butterfly2	8.23	7.74	7.68	7.64	7.65	7.73	7.68	7.40	7.71	7.67	7.40	7.34	7.33	7.46	7.39
Butterfly3	3.61	3.41	3.37	3.44	3.44	3.41	3.37	3.27	3.34	3.32	3.27	3.25	3.25	3.36	3.31
Butterfly4	3.61	3.39	3.34	3.42	3.42	3.39	3.34	3.24	3.31	3.29	3.23	3.23	3.23	3.34	3.29
Butterfly5	3.46	3.21	3.30	3.30	3.30	3.21	3.30	3.16	3.21	3.21	3.16	3.15	3.15	3.17	3.26
Butterfly6	5.84	5.44	5.48	5.50	5.50	5.45	5.48	5.25	5.39	5.38	5.26	5.21	5.22	5.30	5.35
Butterfly7	3.71	3.48	3.45	3.54	3.54	3.48	3.45	3.31	3.40	3.38	3.31	3.31	3.30	3.44	3.38
Butterfly8	4.27	4.05	3.95	4.03	4.03	4.05	3.95	3.87	3.98	3.95	3.87	3.85	3.84	3.98	3.85
Colorchart	3.26	3.04	3.04	3.11	3.11	3.04	3.04	2.95	2.97	2.97	2.95	2.94	2.93	3.04	3.01
CD	2.31	2.21	2.23	2.23	2.23	2.21	2.23	2.17	2.21	2.21	2.17	2.18	2.17	2.20	2.20
Cloth2	6.08	5.79	5.82	5.66	5.66	5.79	5.82	5.51	5.79	5.78	5.51	5.46	5.51	5.48	5.63
Cloth3	5.15	4.96	4.93	4.93	4.93	4.96	4.93	4.85	5.02	5.00	4.86	5.00	4.91	5.00	4.88
Cloth6	5.68	5.35	5.41	5.38	5.39	5.35	5.41	5.25	5.41	5.40	5.25	5.32	5.31	5.33	5.36
Flower	4.86	4.61	4.60	4.62	4.62	4.61	4.60	4.49	4.59	4.57	4.49	4.47	4.47	4.52	4.53
Flower2	5.05	4.73	4.78	4.80	4.80	4.73	4.79	4.60	4.71	4.70	4.61	4.60	4.60	4.64	4.71
Flower3	3.81	3.60	3.62	3.61	3.61	3.60	3.62	3.52	3.60	3.59	3.52	3.50	3.50	3.52	3.55
Party	5.86	5.51	5.61	5.59	5.59	5.51	5.61	5.45	5.51	5.51	5.46	5.45	5.45	5.46	5.56
Tape	5.82	5.52	5.44	5.44	5.44	5.51	5.44	5.25	5.45	5.44	5.26	5.30	5.22	5.43	5.21
Tape2	7.93	7.34	7.32	7.26	7.27	7.34	7.32	7.01	7.30	7.27	7.02	6.92	6.93	7.04	7.01
Tshirts2	8.36	7.77	7.75	7.69	7.7	7.77	7.75	7.44	7.73	7.7	7.45	7.35	7.36	7.47	7.44
**Average**	4.95	4.66	4.66	4.67	4.67	4.66	4.66	4.50	4.63	4.61	4.50	4.49	**4.49**	4.56	4.55

## Data Availability

No new data were created.
